# BHLHE40 Maintains the Stemness of PαS Cells In Vitro by Targeting *Zbp1* through the Wnt/β-Catenin Signaling Pathway

**DOI:** 10.3390/biomedicines11082190

**Published:** 2023-08-03

**Authors:** Menglong Hu, Yueming Tian, Xuenan Liu, Qian Guo, Dazhuang Lu, Xu Wang, Longwei Lv, Xiao Zhang, Yunsong Liu, Yongsheng Zhou, Ping Zhang

**Affiliations:** 1 Department of Prosthodontics, Peking University School and Hospital of Stomatology, 22 Zhongguancun South Avenue, Haidian District, Beijing 100081, China; dental_hu@bjmu.edu.cn (M.H.); tym00007@163.com (Y.T.); pkukqlxn@163.com (X.L.); bjmukqxfgq@163.com (Q.G.); ludazhuang666@gmail.com (D.L.); wangxu0315@bjmu.edu.cn (X.W.); lvlongwei1227@126.com (L.L.); zx8213163@163.com (X.Z.); liuyunsong@hsc.pku.edu.cn (Y.L.); 2 National Center for Stomatology & National Clinical Research Center for Oral Diseases & National Engineering Research Center of Oral Biomaterials and Digital Medical Devices & Beijing Key Laboratory of Digital Stomatology, 22 Zhongguancun South Avenue, Haidian District, Beijing 100081, China

**Keywords:** BHLHE40, culture in vitro, PαS cells, stemness, Wnt/β-catenin signaling pathway, *Zbp1*

## Abstract

Primary bone mesenchymal stem cells (BMSCs) gradually lose stemness during in vitro expansion, which significantly affects the cell therapeutic effects. Here, we chose murine PαS (SCA-1^+^PDGFRα^+^CD45^−^TER119^−^) cells as representative of BMSCs and aimed to explore the premium culture conditions for PαS cells. Freshly isolated (fresh) PαS cells were obtained from the limbs of C57/6N mice by fluorescence-activated cell sorting (FACS). We investigated the differences in the stemness of PαS cells by proliferation, differentiation, and stemness markers in vitro and by ectopic osteogenesis and chondrogenesis ability in vivo, as well as the changes in the stemness of PαS cells during expansion in vitro. Gain- and loss-of-function experiments were applied to investigate the critical role and underlying mechanism of the basic helix–loop–helix family member E40 (BHLHE40) in maintaining the stemness of PαS cells. The stemness of fresh PαS cells representative in vivo was superior to that of passage 0 (P0) PαS cells in vitro. The stemness of PαS cells in vitro decreased gradually from P0 to passage 4 (P4). Moreover, BHLHE40 plays a critical role in regulating the stemness of PαS cells during in vitro expansion. Mechanically, BHLHE40 regulates the stemness of PαS cells by targeting *Zbp1* through the Wnt/β-catenin signaling pathway. This work confirms that BHLHE40 is a critical factor for regulating the stemness of PαS cells during expansion in vitro and may provide significant indications in the exploration of premium culture conditions for PαS cells.

## 1. Introduction

Bone mesenchymal stem cells (BMSCs) have been widely used in bone tissue engineering and stem cell therapy due to their good proliferative capacity and strong multidirectional differentiation [1,2]. However, only approximately 1/100,000 bone marrow monocytes in vivo are BMSCs; therefore, in vitro culture expansion is required to obtain sufficient BMSCs [3]. Unfortunately, the stemness of BMSCs may decrease during in vitro culture and expansion, leading to reduced effectiveness of BMSCs in regenerative tissue engineering and cell therapy [4,5]. Therefore, maintaining the stemness of BMSCs during in vitro expansion is key for effective stem cell therapy [6]. In addition, the biological characteristics of in vivo BMSCs may differ considerably from those of BMSCs cultured in vitro, although differences in stemness have not been investigated [7,8]. Understanding differences in stemness between in vivo and in vitro BMSCs would be of great help in maintaining the stemness of BMSCs during in vitro expansion.

BMSCs are heterogeneous and include different cell subsets that have different functions [9]. Therefore, cell subsets with strong proliferative capacity and multidirectional differentiation are more suitable for investigating the stemness of BMSCs during in vitro expansion culture [10]. Mouse PαS cells (PDGFRα^+^SCA-1^+^CD45^−^TER119^−^) are a subset of mouse BMSCs, with strong proliferative potential and trilineage differentiation capacity [11]. In addition, some studies have noted that PαS cells have similar homology with human BMSCs, which can reflect the status of human BMSCs to a certain extent [11,12].

Basic helix–loop–helix family member E40 (BHLHE40) is a transcription factor that is highly conserved in mammalian species [13]. Previous research showed that BHLHE40 is involved in a variety of basic cellular processes, which included cell proliferation, cell differentiation, cell cycle regulation, metabolism, apoptosis, and immune regulation [13,14,15,16,17,18,19]. For example, studies have shown that BHLHE40 either promotes or inhibits cell proliferation and regulates the production of adipose tissue [20,21,22,23]. In addition, BHLHE40 promotes the metastasis of liver tumor cells and the growth of gastric tumor cells and breast tumor cells [24,25,26,27,28,29]. Such studies indicate that BHLHE40 plays a critical role in cell biology, however, the role of BHLHE40 in maintaining stemness in PαS cells is still unclear.

**Figure 1:**
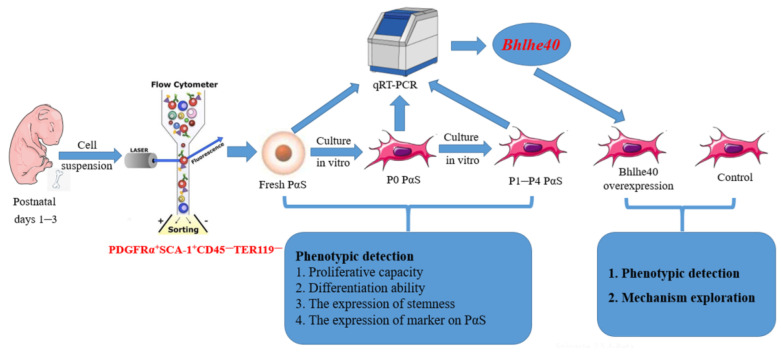
Schematic diagram of the whole research process.

In this study, PαS cells were chosen to represent BMSCs. The research process is shown in Figure 1. We found that the expression of stemness genes and surface markers, as well as the ectopic osteogenic differentiation capacity, were significantly higher in freshly isolated (Fresh) PαS cells than in passage 0 (P0) PαS cells. In addition, the expression of stemness and surface marker genes in PαS cells decreased gradually from P0 to passage 4 (P4). The proliferation and differentiation capacities of PαS cells also decreased gradually from P0 to P4 both in vitro and in vivo. Moreover, we noted that the mRNA expression level of *Bhlhe40* not only in fresh PαS cells was significantly lower than in P0 PαS cells, but also decreased gradually from P0 to P4 culture in vitro. Most importantly, overexpression of *Bhlhe40* promoted cell proliferation, as well as osteogenic and chondrogenic differentiation of PαS cells in vitro. Mechanically, BHLHE40 activated Z-DNA binding protein 1 (*Zbp1*) to regulate the osteogenic and chondrogenic differentiation potential of PαS cells through Wnt/β-catenin signaling.

Collectively, our present work provided significant indication for exploration of better culture conditions for PαS cells. Small molecule drugs that stimulate BHLHE40 expression may be developed to maintain the stemness of PαS cells when they are expanded and cultured in vitro.

## 2. Materials and Methods

The specific information of animals, materials, and reagents used in the present study is listed in Table S1.

### 2.1. Cell Culture

Fresh PαS cells were extracted from the limbs of C57BL/6N mice 1 to 3 days after birth, which were not inoculated on the well plate. The method used is described by Matsuzaki and Zeller et al. [11,30]. The main steps were to extract the limbs of C57/6N mice, cut the limbs in the tube, decompose them with collagenase I and II at 37 °C, filter, break the red blood cells, incubate the flow antibody, and then to obtain the fresh PαS cells (SCA-1^+^PDGFRα^+^CD45^−^TER119^−^) through fluorescence-activated cell sorting (FACS). Fresh PαS cells were inoculated on a well plate to obtain P0 PαS cells, and passage 1 (P1) to P4 PαS cells were also obtained by digestion and passage in the well plate.

PαS cells were cultured in a proliferation medium that consisted of minimum essential medium α, fetal bovine serum (10%), penicillin G (100 U/mL), and streptomycin (100 mg/mL). Osteogenic medium was created by supplementing the proliferation medium with L-ascorbic acid (0.2 mM), β-glycerophosphoric acid (10 mM), and dexamethasone (100 nM). Chondrogenic medium was created by supplementing the proliferation medium with L-lactic acid diphosphate (1 µM), sodium pyruvate (0.1%), dexamethasone (100 nM), insulin-transferrin-selenium (1%), L-proline (40 µg/mL), and transforming growth factor-β1 (10 ng/mL). The cell culture conditions were 95% air, 5% CO_2_, and 37 °C [31].

### 2.2. Lentivirus Infection, RNA Transfection and Use of Inhibitor of Wnt/β-Catenin Signaling Pathway

*Bhlhe40*-overexpressing lentivirus (Bhlhe40) and negative control (Vector) were purchased from GenePharma. To construct cell lines with stable *Bhlhe40* overexpression, lentivirus suspension containing polystyrene (5 mg/mL) was added to the cell proliferation medium when the fusion rate of P1 or Passage 2 (P2) PαS cells reached 50 to 70%. After 72 to 96 h of lentivirus infection, the cells were screened using puromycin (1 µg/mL). Passage 3 (P3) or P4 PαS cells with stable mRNA overexpression of *Bhlhe40* were used in subsequent experiments.

The short interference (si) RNA sequences used to target *Zbp1* (si*Zbp1*) and the negative control (siNC) are listed in Table 1. The siRNAs were transfected using Lipofectamine 3000, in accordance with the manufacturer’s instructions. Cells were collected 48 h after transfection, to analyze gene expression. During differentiation induction, transfection was repeated every 2 to 3 days. To investigate the signal pathway mechanisms, the cell media were supplemented with IWR-1 endo (10 µM), which is a Wnt/β-catenin signaling pathway inhibitor.

### 2.3. Cell Proliferation Capacity Test

Initially, 10^3^ cells were seeded in a 96-well plate and the culture medium changed every 2 to 3 days. Cell Counting Kit-8 (CCK8) proliferation assay was performed in accordance with the manufacturer’s instructions.

Single cells were added to 96-well plates where media were added, using the FACS apparatus. In addition, each well of the 6-well plate was seeded with 500 cells. And the media were changed every 2 to 3 days. On day 14, the culture media were removed from the plates and the cells were washed by phosphate-buffered saline (PBS). Then, the cells were fixed with paraformaldehyde (4%) and stained with crystal violet (1%). Finally, the 96-well plates and 6-well plate were scanned and photographed.

### 2.4. Alkaline Phosphatase (ALP) Staining and Activity

PαS cells were seeded in 12-well plates; the cells were fixed in cold ethanol and washed with PBS after 7 days of osteogenic induction. ALP staining and quantification were performed in accordance with the manufacturer’s instructions. We used an ALP staining and detection kit and a bicinchoninic acid assay protein detection kit. ALP-stained images were scanned and the absorbance of ALP quantification solution was measured. Total protein concentrations were measured after normalization.

### 2.5. Alizarin Red S (ARS) Staining and Quantification

PαS cells were inoculated in 12-well plates, the cells were fixed in ethanol and rinsed with distilled water after 14 days of osteogenic induction. Then, cells were stained with ARS solution (2%, pH 4.2), and ARS-stained images were scanned. The plates were incubated with cetylpyridine chloride (100 mM), and the absorbance of the ARS quantification solution was measured.

### 2.6. PαS Cells in Well Plates Stained with Alcian Blue

PαS cells were seeded in 12-well plates. After 21 days of chondrogenic induction, PαS cells were fixed with ethanol, washed with PBS, stained with Alcian blue solution (pH 2.5), and photographed under a microscope [32].

### 2.7. Chondrosphere Sections Stained with Alcian Blue and Sirius Red

After digestion, PαS cells were counted and resuspended at 5 × 10^5^ cells in a 15 mL centrifugal tube and centrifuged (1000 rpm, 5 min) to collect the cells. Next, chondrogenic induction medium was added and, after 21 days, the cell pellets were rinsed with PBS and fixed in paraformaldehyde (4%) overnight. Finally, the chondrospheres were then stained with Alcian blue and Sirius red.

### 2.8. RNA Extraction and Quantitative Reverse Transcription-Polymerase Chain Reaction (qRT-PCR)

PαS cells were inoculated in 6-well plates and total RNA was extracted by TRIzol reagent. A Nano Drop 8000 spectrophotometer was used to measured the RNA concentrations. In accordance with the manufacturer’s instructions, total RNA was reverse transcribed into single-stranded cDNA by the Prime Script RT Reagent Kit, and qRT-PCR reactions were performed using Power SYBR Green PCR Master Mix. Glyceraldehyde 3-phosphate dehydrogenase (*Gapdh*) was used as an internal reference, and gene expression was normalized. The gene expression was analyzed by the 2^−ΔΔCT^ method. The mouse primers used in the present study are listed in Table 1.

**Table 1:** Sequences of siRNA and primers used in this study.

Name	Sense Strand/Sense Primer (5′–3′)	Antisense Strand/Antisense Primer (5′–3′)
si-RNA		
si-*Zbp1*	GCCUGCAACAUGGAGCAUATT	UAUGCUCCAUGUUG-CAGGCTT
Si-NC	UUCUCCGAACGUGUCACGUTT	ACGUGACACGUUCGGAGAATT
Primers		
m*Gapdh*	TGGCCTTCCGTGTTCCTAC	GAGTTGCTGTTGAAGTCGCA
m*Alp*	CCAACTCTTTTGTGCCAGAGA	GGCTACATTGGTGTTGAGCTTTT
m*Runx2*	AGAGTCAGATTACAGATCCCAGG	TGGCTCTTCTTACTGA-GAGAGG
m*Nanog*	AGGACAGGTTTCAGAAGCAGA	CCATTGCTAG-TCTTCAACCACTG
m*Sox2*	GCGGAGTGGAAACTTTTGTCC	GGGAAGCGTGTACTTATCCTTCT
m*Oct4*	AGAGGATCACCTTGGGGTACA	CGAA-GCGACAGATGGTGGTC
m*Pdgfrα*	TATCCTCCCAAACGAGAATGAGA	GTGGTTGTAGTAG-CAAGTGTACC
m*Sca-1*	CTGACTGGAAAGCCGAAACTC	CGACCCGTCCTTT-GAATTTCT
m*Bhlhe40*	CTGTCAGGGATGGATTTTGCC	GCTGTCTTCGCTCCGTTTTATTC
m*Bglap*	CTGACCTCACAGATCCCAAGC	TGGTCTGATAGCTCGTCACAAG
m*Col1a1*	TAAGGGTCCCCAATGGTGAGA	GGGTCCCTCGACTCC-TACAT
m*Acan*	CCTGCTACTTCATCGACCCC	AGATGCTGTTGACTCGAACCT
m*Sox9*	CGGAACAGACTCACATCTCTCC	GCTTGCACGTCGGTTTTGG
m*Comp*	ACTGCCTGCGTTCTAGTGC	CGCCGCATTAGTCTCCTGAA
m*Zbp-1*	AAGAGTCCCCTGCGATTATTTG	TCTGGATGGCGTTT-GAATTGG
m*Wnt3a*	CTCCTCTCGGATACCTCTTAGTG	GCATGATCTCCACGTAG-TTCCTG
m*Ctnnb1*	ATGGAGCCGGACAGAAAAGC	CTTGCCACTCAGGGAAGGA
m*Ccnd1*	GCGTACCCTGACACCAATCTC	CTCCTCTTCGCACTTCTGCTC
m*Spp1*	AGCAAGAAACTCTTCCAAGCAA	GTGAGATTCGTCAGAT-TCATCCG

### 2.9. Enzyme Linked Immunosorbent Assay (ELISA)

After the PαS cells were rinsed twice with PBS, a cell scraper was used to carefully detach the cells. Next, the culture medium was centrifuged (3000 rpm, 10 min) and the supernatant was discarded. ELISAs were performed with reference to the kit manufacturer’s instructions. The optical density of each well was measured against a blank control well at 450 nm using a microplate reader. Protein concentrations of the samples were calculated using a standard curve [33].

### 2.10. Chromatin Immunoprecipitation (ChIP) Assay

ChIP assays were performed as described previously [34]. Soluble chromatin was diluted and immunoprecipitated with a BHLHE40 antibody. After crosslinks were removed, the immune complexes containing the DNA were purified and eluted. The precipitated DNA was analyzed by qRT-PCR. The ChIP primer sequences for *Zbp1* were 5′–ATTGTGAGCTGAGTGGTGGA–3′ (forward) and 5′–TTCTCAGTGACCCGTGAACA–3′ (reverse).

### 2.11. Ectopic Osteogenesis and Chondrogenesis In Vivo

Ectopic osteogenic transplantation of PαS cells and lentivirus-infected PαS cells (Bhlhe40 or Vector) was investigated in nude mice. After trypsinization and resuspension, cells were mixed with β-tricalcium phosphate granules. The complex was subcutaneously implanted into the dorsal space of BALB/C mice (6-week-old, *n* = 5 per group). Considering animal ethics and the reliability of experimental results, 5 mice were selected in each group [34,35,36]. After 8 weeks, samples were collected, fixed with paraformaldehyde (4%) for 24 h, and decalcified in ethylenediamine-tetra-acetic acid (10%, pH 7.4) for 14 days. The samples were then subjected to follow-up treatment and stained with hematoxylin and eosin (H&E), Masson’s trichome, and immunohistochemistry (IHC)–osteocalcin (OCN). In addition, in the results of H&E, Masson’s trichome, and IHC-OCN staining, the area proportion of newly formed lamellar bone was compared and analyzed. If there was no lamellar bone formation, the homogeneous stained areas that represented the new bone were compared.

Ectopic chondrogenic transplantation of PαS cells and lentivirus-infected PαS cells (Bhlhe40 or Vector) was also investigated in nude mice. After 7 days of chondrogenic induction, PαS cells were collected and mixed with collagen membrane, and the complex was also subcutaneously implanted into the dorsal space of BALB/C mice (6-week-old, *n* = 5 per group). After 4 weeks, samples were collected and fixed with paraformaldehyde (4%) for 24 h. The samples were then subjected to follow-up treatment and stained with Alcian blue and Sirius red. Moreover, in the results of Alcian blue and Sirius red stained, the homogeneous stained areas that represented the new chondrogenic were compared.

### 2.12. Statistical Analyses

SPSS software (ver. 25.0; IBM Corp., Armonk, NY, USA) was used for statistical analyses in this study. The Q–Q plots and Shapiro–Wilk tests were used to evaluate the distribution of measured data, which indicated the normality of the data in this study. For normally distributed data, the Student’s *t*-test was used to analyze the differences between two groups, and one-way analysis of variance (ANOVA) was used for comparisons between more than two groups, followed by Tukey’s post hoc test. For data with obvious asymmetry, the non-parametric Kruskal–Wallis test was used in this study. All values in the present study are shown as means ± standard deviation for three independent experiments per group [37,38,39,40,41]. *p* < 0.05 was considered statistically significant (* *p* < 0.05; ** *p* < 0.01; *** *p* < 0.001).

## 3. Results

### 3.1. The Stemness of Fresh PαS Cells Was Superior to That of In Vitro P0 Cells

**Figure 2:**
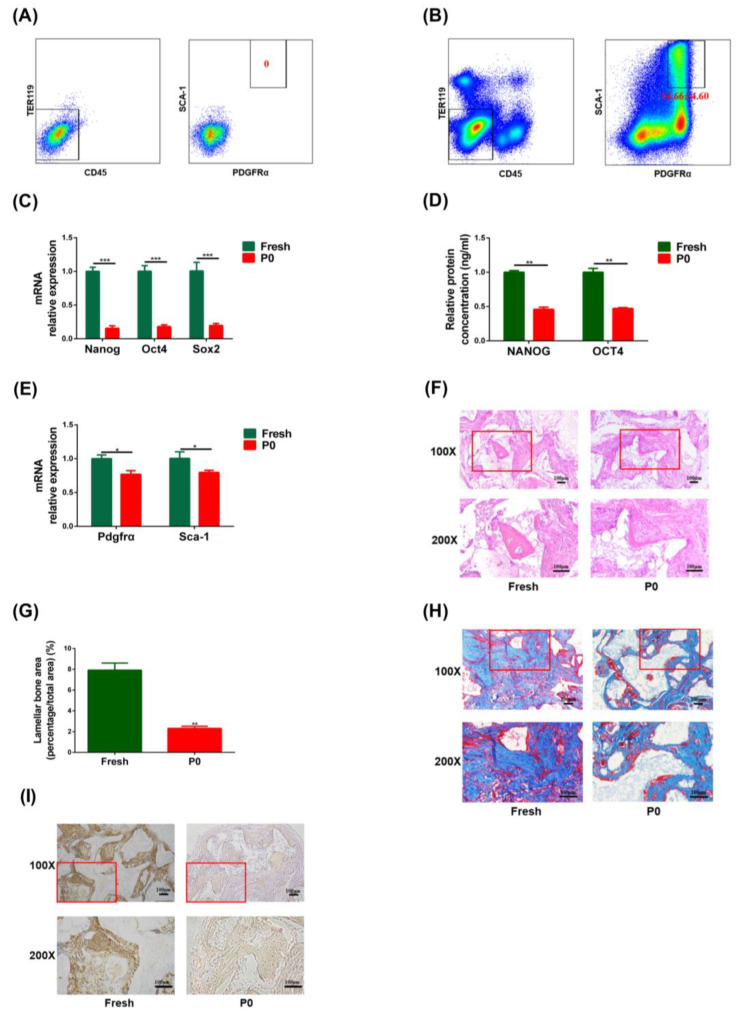
The stemness of freshly isolated (Fresh) PαS cells representative in vivo was superior to that of P0 PαS cells in vitro. (**A**,**B**) Fresh PαS cells from C57/6N mice were obtained by FACS. (**C**) Relative expression of *Nanog*, *Oct4*, and *Sox2* mRNA was evaluated by qRT-PCR in fresh and P0 PαS cells. *Gapdh* was used for normalization. (**D**) Relative expression of NANOG and OCT4 protein was evaluated by ELISA in fresh and P0 PαS cells. (**E**) Relative expression of *Pdgfrα* and *Sca-1* mRNA was evaluated by qRT-PCR in fresh and P0 PαS cells. *Gapdh* was used for normalization. (**F**,**H**,**I**) Ectopic bone formation of fresh and P0 PαS cells in vivo was evaluated by H&E, Masson’s trichome, and IHC-OCN staining assays. Scale bar = 100 μm, *n* = 3. (**G**) Quantitative analysis of new lamellar bone by H&E staining of fresh and P0 PαS cells. Data are represented as mean ± SD of three independent experiments. * *p* < 0.05; ** *p* < 0.01; *** *p* < 0.001, Student’s *t*-test and one-way ANOVA. P0, passage 0; FACS, fluorescence activated cell sorting; *Gapdh*, glyceraldehyde 3-phosphate dehydrogenase; OCT4, organic cation/carnitine transporter4; NANOG, nanog homeobox; *Sox2*, sex determining region Y-box 2; ELISA, enzyme linked immunosorbent assay; *Pdgfrα*, platelet derived growth factor receptor, alpha polypeptide; qRT-PCR, quantitative reverse transcription polymerase chain reaction; H&E, hematoxylin and eosin; IHC, immunohistochemical; OCN, osteocalcin; ANOVA, analysis of variance.

The flow sorting results for the C57/6N fresh PαS cells are shown in Figure 2A,B. Approximately 2 to 3 million PαS cells were obtained from the limbs of 20 C57/6N mice 1 to 3 days after birth, and the proportion of PαS cells obtained was 14.66% ± 4.60%.

qRT-PCR results displayed the expression level of the stemness genes (*Oct4*, *Sox2*, and *Nanog*) and the surface marker genes (*Pdgfrα* and *Sca-1*) were significantly lower in P0 PαS cells than in fresh PαS cells (Figure 2C,E). The ELISA results demonstrated that protein expression of the OCT4 and NANOG was also significantly lower in P0 PαS cells than in fresh PαS cells (Figure 2D).

H&E staining showed that there was new eosinophilic homogeneous red-stained lamellar bone in both the Fresh and P0 groups; the structure of this bone resembled lacunae, but there was significantly more new lamellar bone in the Fresh group than in the P0 group (Figure 2F). This finding was confirmed by quantitative analysis of the new lamellar bone tissue (Figure 2G). Furthermore, Masson’s trichome and IHC staining showed that there were significantly more blue-stained collagen fibers or more OCN staining (brown) in the cytoplasm and interstitia in the Fresh group than in the P0 group (Figure 2H,I). These data demonstrated that freshly isolated PαS cells possessed great differentiation potential compared with P0 cells.

### 3.2. The Proliferative Capacity, Expression of Stemness Genes and Cell Surface Markers in PαS Cells Decreased Gradually from P0 to P4

**Figure 3:**
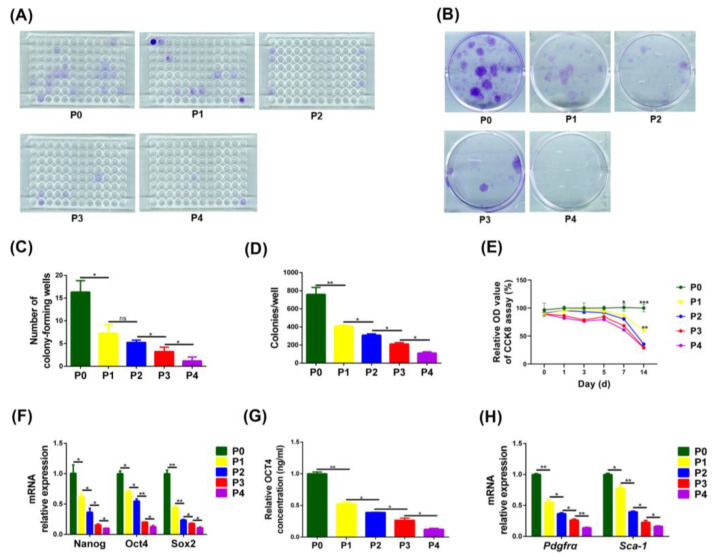
Proliferative capacity and expression of stemness genes and cell-surface markers in PαS cells decreased gradually from P0 to P4 in vitro. (**A**,**C**) Results of a single-cell clone formation assay for PαS cells from P0 to P4 in vitro. (**B**,**D**) Results of a multicellular clone formation assay for PαS cells from P0 to P4 in vitro. (**E**) Relative OD value of the CCK8 assay (%) for PαS cells from P0 to P4 in vitro. (**F**) Relative expression of *Nanog*, *Oct4*, and *Sox2* mRNA was evaluated by qRT-PCR in PαS cells from P0 to P4 in vitro. *Gapdh* was used for normalization. (**G**) Relative expression of OCT4 protein was evaluated by ELISA in PαS cells from P0 to P4 in vitro. (**H**) Relative expression of *Pdgfrα* and *Sca-1* mRNA was evaluated by qRT-PCR in PαS cells from P0 to P4 in vitro. *Gapdh* was used for normalization. Data are represented as mean ± SD of three independent experiments. * *p* < 0.05; ** *p* < 0.01; *** *p* < 0.001; ns *p* > 0.05, Student’s *t*-test and one-way ANOVA. P0, passage 0; P4, passage 4; OD, optical density; CCK8, cell counting kit-8; *Oct4*, organic cation/carnitine transporter4; *Nanog*, nanog homeobox; *Sox2*, sex determining region Y-box 2; *Gapdh*, glyceraldehyde 3-phosphate dehydrogenase; ELISA, enzyme linked immunosorbent assay; *Pdgfrα*, platelet derived growth factor receptor, alpha polypeptide; qRT-PCR, quantitative reverse transcription polymerase chain reaction; ANOVA, analysis of variance.

The single-cell and multicellular clone formation capacity of PαS cells also decreased gradually from P0 to P4 (Figure 3A,D). In addition, the CCK8 proliferation assay displayed a significant difference in proliferative capacity between PαS cells at P0 and PαS cells at P1 to P4 at 14 days, and between cells at P1 and P2 to P4 at 14 days, but there was no significant difference among PαS cells at P2 to P4 at 14 days (Figure 3E).

The qRT-PCR results showed the expression of the stemness genes and surface marker genes in PαS cells decreased gradually from P0 to P4 (Figure 3F,H). Furthermore, the ELISA results indicated the expression of the OCT4 in PαS cells decreased gradually from P0 to P4 (Figure 3G). All the data indicated that the proliferative capacity, expression of stemness genes and cell surface markers in PαS cells decreased gradually from P0 to P4.

### 3.3. The Osteogenic and Chondrogenic Differentiation Capacity of PαS Cells Decreased Gradually from P0 to P4

**Figure A1:**
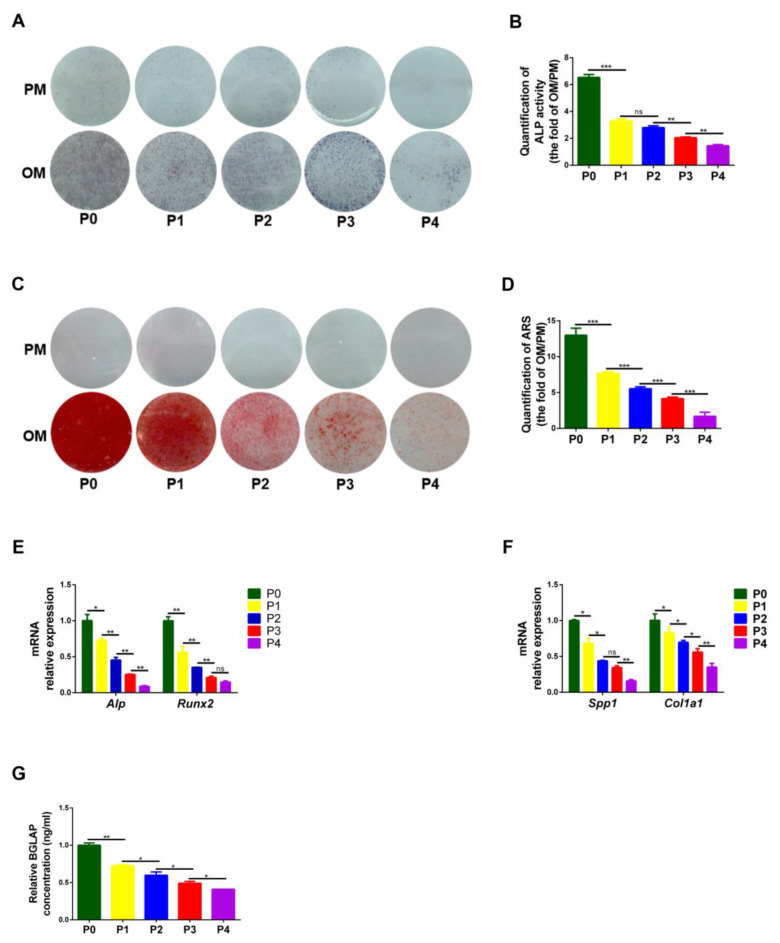
The osteogenic differentiation capacity of PαS cells decreased gradually from P0 to P4 in vitro. (**A**,**B**) ALP staining and activity of PαS cells from P0 to P4 in vitro. (**C**,**D**) ARS staining and quantification of PαS cells from P0 to P4 in vitro. (**E**) Relative expression of *Alp* and *Runx2* mRNA was evaluated by qRT-PCR in PαS cells from P0 to P4 in vitro after 7 days of osteogenic differentiation. *Gapdh* was used for normalization. (**F**) Relative expression of *Spp1* and *Col1a1* mRNA was evaluated by qRT-PCR in PαS cells from P0 to P4 in vitro after 14 days of osteogenic differentiation. *Gapdh* was used for normalization. (**G**) Relative expression of BGLAP protein was evaluated by ELISA in PαS cells from P0 to P4 in vitro after 14 days of osteogenic differentiation. Data are represented as mean ± SD of three independent experiments. * *p* < 0.05; ** *p* < 0.01; *** *p* < 0.001; ns *p* > 0.05.

After 7 or 14 days of osteogenic induction, ALP staining and activity in PαS cells decreased gradually from P0 to P4 (Figure A1A,B). ARS staining and quantitative PαS cells showed a consistent trend (Figure A1C,D). In addition, the mRNA expression of the *Alp*, *Runx2*, *Spp1*, *Col1a1* as well as the protein expression of BGLAP in PαS cells all decreased gradually from P0 to P4 (Figure A1E,G).

**Figure A2:**
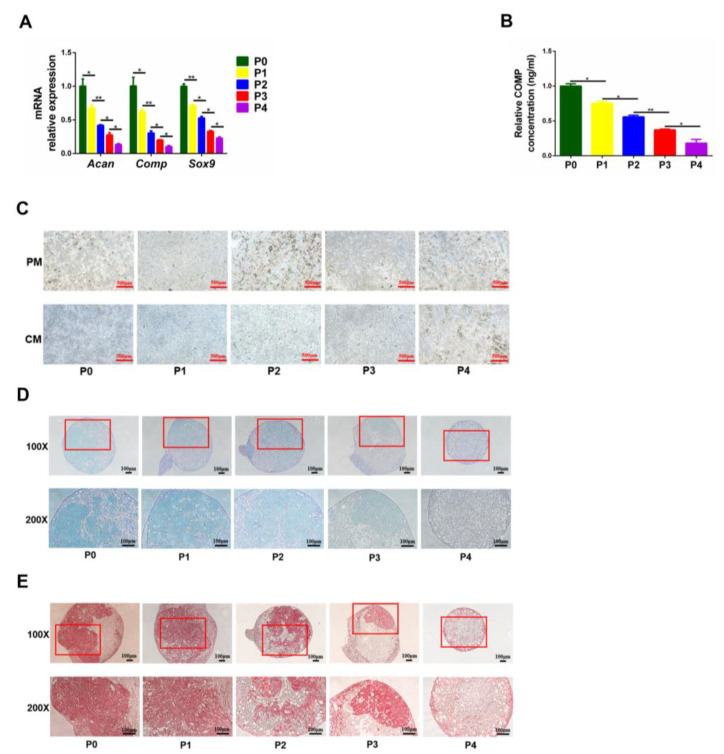
The chondrogenic differentiation capacity of PαS cells decreased gradually from P0 to P4 in vitro. (**A**) Relative expression of *Acan*, *Comp*, and *Sox9* mRNA was evaluated by qRT-PCR in PαS cells from P0 to P4 in vitro after 21 days of chondrogenic differentiation. *Gapdh* was used for normalization. (**B**) Relative expression of COMP protein was evaluated by ELISA in PαS cells from P0 to P4 in vitro after 21 days of chondrogenic differentiation. (**C**) Alcian blue staining in well plates of PαS cells from P0 to P4 in vitro after 21 days of chondrogenic differentiation. Scale bar = 500 μm, *n* = 3. (**D**,**E**) Alcian blue and Sirius red staining of chondrosphere sections of PαS cells from P0 to P4 in vitro after 21 days of chondrogenic differentiation. Scale bar = 100 μm, *n* = 3. Data are represented as mean ± SD of three independent experiments. * *p* < 0.05; ** *p* < 0.01; ns *p* > 0.05.

After 21 days of chondrogenic induction, the mRNA expression of the *Acan*, *Sox9*, and *Comp* and the protein expression of COMP in PαS cells all showed a decreased trend (Figure A2A,B). In addition, the Alcian blue staining in well plates and the Alcian blue and Sirius red staining of chondrosphere sections all showed gradually less staining of PαS cells from P0 to P4 (Figure A2C,E).

**Figure 4:**
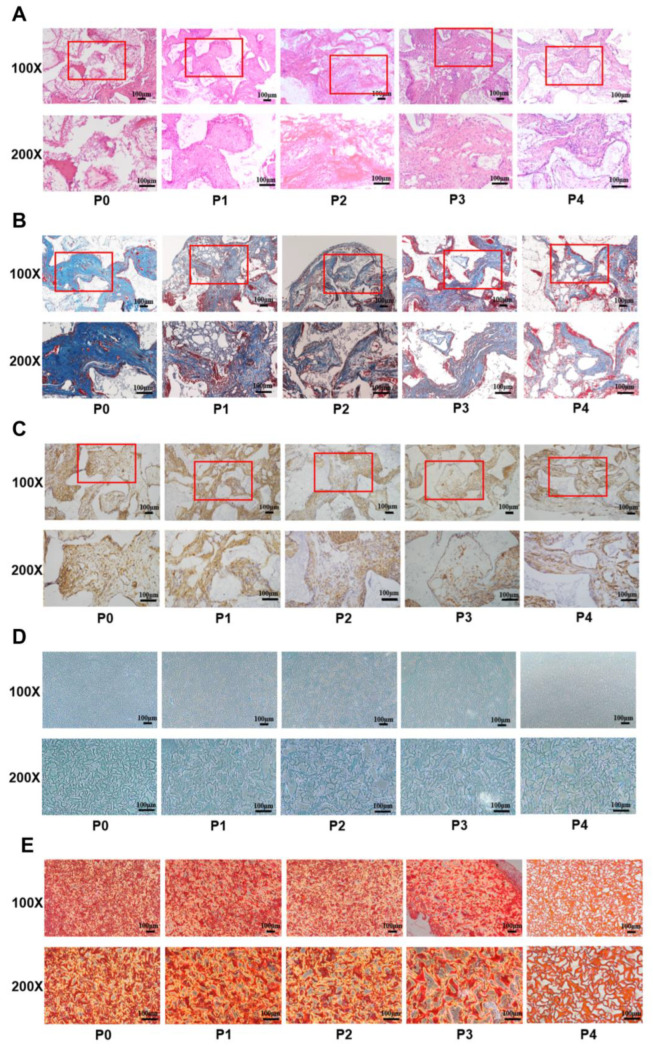
The osteogenic and chondrogenic differentiation capacity of PαS cells decreased gradually from P0 to P4 in vivo. (**A**–**C**) Ectopic bone formation of PαS cells from P0 to P4 in vivo was evaluated by H&E, Masson’s trichome, and IHC-OCN staining assays. Scale bar = 100 μm, *n* = 3. (**D**,**E**) Ectopic cartilage formation of PαS cells from P0 to P4 in vivo was evaluated by Alcian blue and Sirius red staining assays. Scale bar = 100 μm, *n* = 3. P0, passage 0; P4, passage 4; H&E, hematoxylin and eosin; IHC, immunohistochemical; OCN, osteocalcin.

H&E staining showed that there was new eosinophilic homogeneous red-stained lamellar bone in the P0 group, but there was no obvious new lamellar bone in groups P1 to P4. In addition, the proportion of eosinophilic red-stained tissue also showed a decreased trend from P0 to P4 (Figure 4A). Moreover, Masson’s trichome staining and the IHC-OCN staining results both showed that the degree of staining decreased gradually from P0 to P4 (Figure 4B,C). Furthermore, the ectopic chondrogenesis staining results in vivo displayed that the proportions of Alcian blue- and Sirius red-stained new cartilage tissue also decreased gradually from P0 to P4 (Figure 4D,E). These data demonstrated that the stemness of PαS cells decreased gradually from P0 to P4.

### 3.4. BHLHE40 Stimulated Proliferation, the Expression of Stemness Genes and Cell Surface Markers in PαS Cells

**Figure 5:**
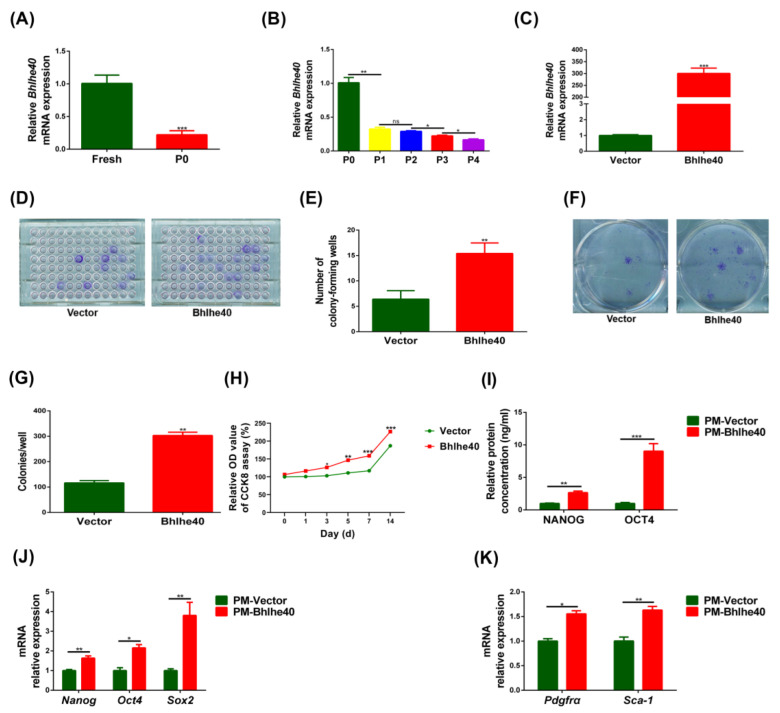
Overexpression of *Bhlhe40* promoted proliferation, expression of stemness genes, and cell surface markers in PαS cells in vitro. (**A**) Relative expression of *Bhlhe40* mRNA was evaluated by qRT-PCR in fresh and P0 PαS cells. *Gapdh* was used for normalization. (**B**) Relative expression of *Bhlhe40* mRNA was evaluated by qRT-PCR in PαS cells from P0 to P4 in vitro. *Gapdh* was used for normalization. (**C**) Relative expression of *Bhlhe40* mRNA in overexpressed-*Bhlhe40* (Bhlhe40) group and negative control (Vector) group in vitro was evaluated by qRT-PCR. *Gapdh* was used for normalization. (**D**,**E**) Results of a single-cell clone formation assay for Bhlhe40 group and Vector group in vitro. (**F**,**G**) Results of a multicellular clone formation assay for Bhlhe40 group and Vector group in vitro. (**H**) Relative OD value of the CCK8 assay (%) for Bhlhe40 group and Vector group in vitro. (**I**) Relative expression of NANOG and OCT4 protein was evaluated by ELISA in Bhlhe40 group and Vector group in vitro. (**J**) Relative expression of *Nanog*, *Oct4*, and *Sox2* mRNA was evaluated by qRT-PCR in Bhlhe40 group and Vector group in vitro. *Gapdh* was used for normalization. (**K**) Relative expression of *Pdgfrα* and *Sca-1* mRNA was evaluated by qRT-PCR in Bhlhe40 group and Vector group in vitro. *Gapdh* was used for normalization. Data are represented as mean ± SD of three independent experiments. * *p* < 0.05; ** *p* < 0.01; *** *p* < 0.001, ns *p* > 0.05, Student’s *t*-test and one-way ANOVA. P0, passage 0; P4, passage 4; OD, optical density; CCK8, cell counting kit-8; *Oct4*, organic cation/carnitine transporter4; *Nanog*, nanog homeobox; *Sox2*, sex determining region Y-box 2; *Gapdh*, glyceraldehyde 3-phosphate dehydrogenase; ELISA, enzyme linked immunosorbent assay; *Pdgfrα*, platelet derived growth factor receptor, alpha polypeptide; qRT-PCR, quantitative reverse transcription polymerase chain reaction; ANOVA, analysis of variance.

**Figure A3:**
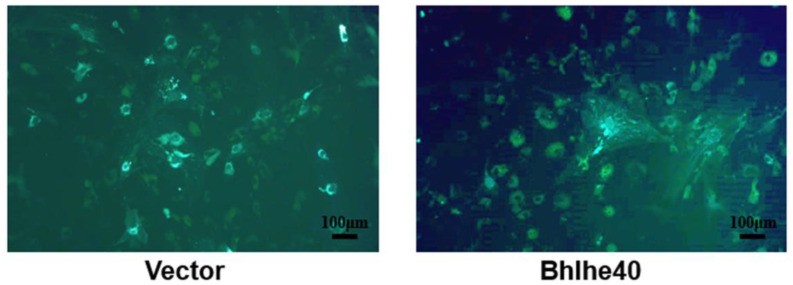
A Lentivirus infection of Bhlhe40 group and Vector group in vitro, observed under an inverted fluorescence microscope. Scale bar = 100 μm, *n* = 3.

In order to explore better culture conditions for freshly isolated cells, we screened transcriptomic changes between primary PαS cells and cultured cells, and detected some changes of expression of transcription factors. qRT-PCR results further verified that Bhlhe40 expression was significantly lower in P0 PαS cells than in fresh PαS cells (Figure 5A). In addition, the mRNA expression of *Bhlhe40* also decreased gradually from P0 to P4, and decreased most significantly from P0 to P1 (Figure 5B). We next infected *Bhlhe40* into P1 and P2 PαS cells; fluorescent staining and qRT-PCR were used to verify the infection efficiency (Figure 5C and Figure A3).

The overexpressed-*Bhlhe40* (Bhlhe40) group exhibited significantly greater potential for single-cell and multicellular clone formation than the negative control (Vector) group (Figure 5D,G). Moreover, the results of CCK8 proliferation assay displayed that there was a significant difference between the Bhlhe40 group and the Vector group from day 3 to day 14 (Figure 5H).

qRT-PCR showed that stemness genes and surface marker genes were significantly higher in the Bhlhe40 group than in the Vector group (Figure 5J,K). In addition, the ELISA results of OCT4, NANOG displayed that the Bhlhe40 group was significantly higher than the Vector group (Figure 5I). All the data indicated that BHLHE40 promoted proliferation, the expression of stemness genes, and cell surface markers of PαS cells.

### 3.5. Overexpression of Bhlhe40 Stimulated Osteogenic and Chondrogenic Differentiation of PαS Cells

**Figure 6:**
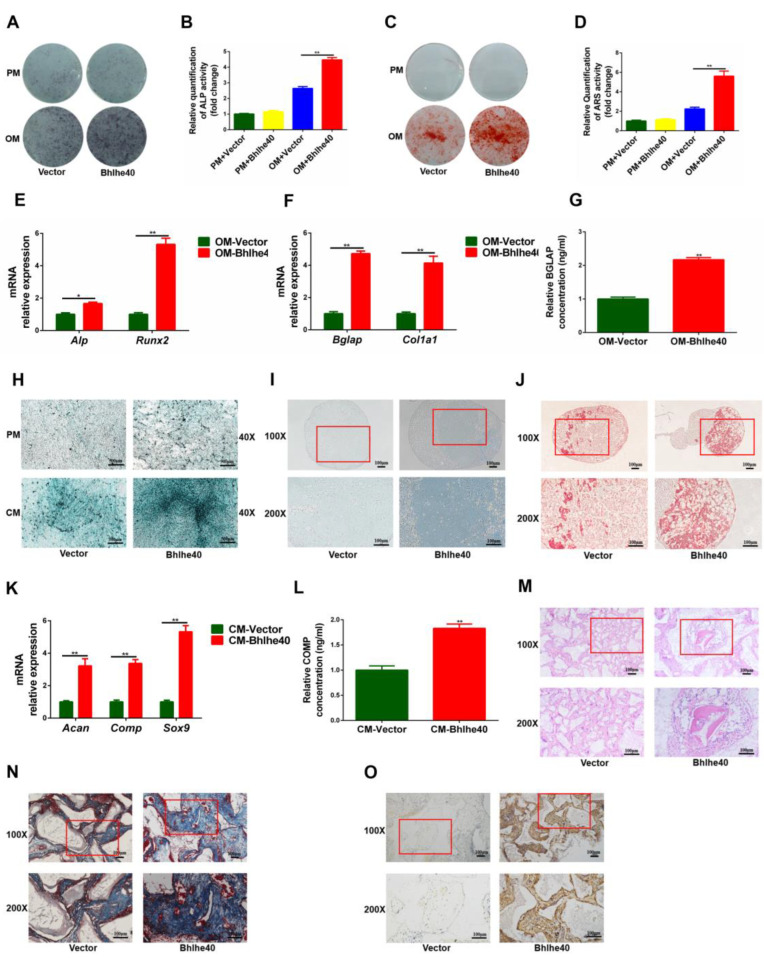
Overexpression of *Bhlhe40* promoted osteogenic and chondrogenic differentiation of PαS cells in vivo and in vitro. (**A**,**B**) ALP staining and activity of overexpressed *Bhlhe40* (Bhlhe40) group and negative control (Vector) group in vitro. (**C**,**D**) ARS staining and quantification of Bhlhe40 group and Vector group in vitro. (**E**) Relative expression of *Alp* and *Runx2* mRNA was evaluated by qRT-PCR in Bhlhe40 group and Vector group in vitro after 7 days of osteogenic differentiation. *Gapdh* was used for normalization. (**F**) Relative expression of *Bglap* and *Col1a1* mRNA was evaluated by qRT-PCR in Bhlhe40 group and Vector group in vitro after 14 days of osteogenic differentiation. *Gapdh* was used for normalization. (**G**) Relative expression of BGLAP protein was evaluated by ELISA in Bhlhe40 group and Vector group in vitro after 14 days of osteogenic differentiation. (**H**) Alcian blue staining in well plate of Bhlhe40 group and Vector group in vitro after 21 days of chondrogenic differentiation. Scale bar = 500 μm, *n* = 3. (**I**,**J**) Alcian blue and Sirius red staining of chondrosphere sections of Bhlhe40 group and Vector group in vitro after 21 days of chondrogenic differentiation. Scale bar = 100 μm, *n* = 3. (**K**) Relative expression of *Acan*, *Comp*, and *Sox9* mRNA was evaluated by qRT-PCR in Bhlhe40 group and Vector group in vitro after 21 days of chondrogenic differentiation. *Gapdh* was used for normalization. (**L**) Relative expression of COMP protein was evaluated by ELISA in Bhlhe40 group and Vector group in vitro after 21 days of chondrogenic differentiation. (**M**–**O**) Ectopic bone formation in Bhlhe40 group and Vector group in vivo was evaluated by H&E, Masson’s trichome, and IHC-OCN staining assays. Scale bar = 100 μm, *n* = 3. Data are represented as mean ± SD of three independent experiments. * *p* < 0.05; ** *p* < 0.01, Student’s *t*-test and one-way ANOVA. ALP, alkaline phosphatase; ARS, alizarin red S; *Runx2*, runt-related transcription factor 2; *Col1a1*, collagen, type I, alpha 1; *Acan*, aggrecan; *Sox9*, sex determining region Y-box 9; BGLAP, bone gamma-carboxyglutamate (gla) protein; COMP, cartilage oligomeric matrix protein; *Gapdh*, glyceraldehyde 3-phosphate dehydrogenase; ELISA, enzyme linked immunosorbent assay; qRT-PCR, quantitative reverse transcription polymerase chain reaction; H&E, hematoxylin and eosin; IHC, immunohistochemical; OCN, osteocalcin; ANOVA, analysis of variance.

After 7 or 14 days of osteogenic induction, ALP staining and activity in the Bhlhe40 group was significantly greater than in the Vector group (Figure 6A,B). In addition, there was a similar trend in ARS staining and quantification (Figure 6C,D). In addition, the mRNA expression of *Alp*, *Runx2*, *Bglap*, *Col1a1* and the protein expression of BGLAP all showed that the Bhlhe40 group was significantly higher than the Vector group (Figure 6E,G).

After 21 days of chondrogenic induction, the results of Alcian blue staining in well plates and the results of Alcian blue and Sirius red staining of chondrosphere sections both showed significantly more staining in the Bhlhe40 group than in the Vector group (Figure 6H,J). Moreover, the qRT-PCR results of the *Acan*, *Sox9*, *Comp* and the ELISA results of COMP were all significantly higher in the Bhlhe40 group than in the Vector group (Figure 6K,L).

H&E staining results showed that the Bhlhe40 group exhibited eosinophilic neoplasia of homogeneous, red-stained lamellar bone, but there was no obvious neoplasia lamellar bone in the Vector group. In addition, the proportion of eosinophilic, red-stained tissue was significantly greater in the Bhlhe40 group than in the Vector group (Figure 6M). Moreover, Masson’s trichome and IHC-OCN staining showed similar results (Figure 6N,O). These data suggested that BHLHE40 promoted stemness of PαS cells.

### 3.6. BHLHE40 Maintains the Stemness of PαS Cells by Activating Zbp1

**Figure 7:**
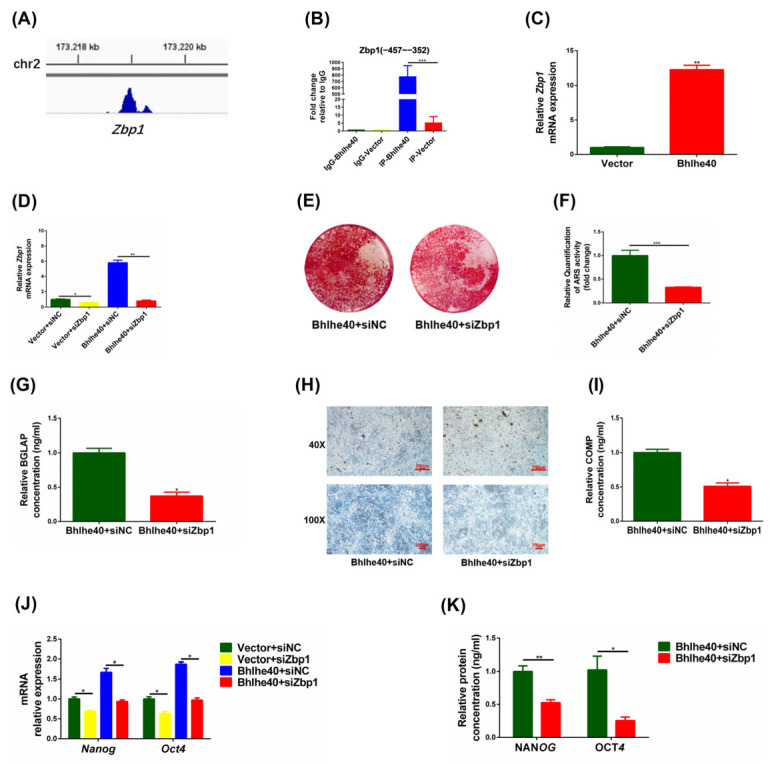
BHLHE40 maintains the stemness of PαS cells by activating *Zbp1*. (**A**) BHLHE40 ChIP-Seq data analysis of C57 mouse cells. The blue shaded part indicates the *Zbp1* enriched region obtained by peak calling. (**B**) ChIP-qPCR results show BHLHE40 bound to *Zbp1* at the promoter region from 457 to 352 bases in overexpressed *Bhlhe40* (Bhlhe40) group compare to negative control (Vector) group. (**C**) Relative expression of *Zbp1* mRNA was evaluated by qRT-PCR in Bhlhe40 group and Vector group in vitro. *Gapdh* was used for normalization. (**D**) Relative expression of *Zbp1* mRNA was evaluated by qRT-PCR in Bhlhe40 group and Vector group transfected with si*Zbp1* and siNC, respectively. *Gapdh* was used for normalization. (**E**,**F**) ARS staining and quantification of Bhlhe40 group and Vector group transfected with si*Zbp1* and siNC after 14 days of osteogenic differentiation, respectively. (**G**) Relative expression of BGLAP protein was evaluated by ELISA in Bhlhe40 + siNC group and Bhlhe40 + siZbp1 in vitro after 14 days of osteogenic differentiation. (**H**) Alcian blue staining in well plates of Bhlhe40 + siNC group and Bhlhe40 + siZbp1 group after 21 days chondrogenic differentiation. Scale bar = 100 μm and 500 μm, *n* = 3. (**I**) Relative expression of COMP protein was evaluated by ELISA in Bhlhe40 + siNC group and Bhlhe40 + siZbp1 in vitro after 21 days of chondrogenic differentiation. (**J**) Relative expression of *Nanog* and *Oct4* mRNA was evaluated by qRT-PCR in Bhlhe40 group and Vector group transfected with si*Zbp1* and siNC, respectively. *Gapdh* was used for normalization. (**K**) Relative expression of NANOG and OCT4 protein was evaluated by ELISA in Bhlhe40 + siNC group and Bhlhe40 + siZbp1 in vitro. Data are represented as mean ± SD of three independent experiments. * *p* < 0.05; ** *p* < 0.01; *** *p* < 0.001, Student’s *t*-test and one-way ANOVA. BHLHE40, basic helix–loop–helix family member E40; ChIP-Seq, chromatin immunoprecipitation sequencing; ARS, alizarin red S; Gapdh, glyceraldehyde 3-phosphate dehydrogenase; si*Zbp1*, small interfering Z-DNA binding protein 1; siNC, short interfering negative control; qRT-PCR, quantitative reverse transcription polymerase chain reaction; ELISA, enzyme linked immunosorbent assay; BGLAP, bone gamma carboxyglutamate protein; COMP, cartilage oligomeric matrix protein; *Nanog*, nanog homeobox; OCT4, organic cation/carnitine transporter4; ANOVA, analysis of variance.

To further investigate the underlying mechanism, we next analyzed the BHLHE40 ChIP-Seq data [42], and summarized the target genes which may be involved in the regulation of stemness (Table 2). Among which, we found that BHLHE40 showed great enrichment for the target gene *Zbp1* (Figure 7A). We next performed ChIP-qPCR to verify the ChIP-seq data, which showed that BHLHE40 bound strongly to *Zbp1*, and the binding region was 457 to 352 bases upstream of the *Zbp1* promoter (Figure 7B). Furthermore, the mRNA expression of *Zbp1* increased significantly when *Bhlhe40* was overexpressed in PαS cells (Figure 7C). Next, qRT-PCR results showed *Zbp1* in the Bhlhe40 + siZbp1 group was significantly lower than in the Bhlhe40 + siNC group, which indicates that si*Zbp1* was successfully transfected into *Bhlhe40*-overexpressing PαS cells (Figure 7D).

After 14 days of osteogenic induction, the ARS staining and quantitative results showed that the Bhlhe40 + siZbp1 group was significantly lower than the Bhlhe40 + siNC group (Figure 7E,F). In addition, the ELISA results of BGLAP showed that the Bhlhe40 + siZbp1 group was significantly less than the Bhlhe40 + siNC group (Figure 7G). After 21 days of chondrogenic induction, the Alcian blue staining in well plates and the ELISA results of COMP both displayed that the Bhlhe40 + siZbp1 group was significantly less than the Bhlhe40 + siNC group (Figure 7H,I). In addition, qRT-PCR and ELISA results both showed that the expression of stemness genes in the Bhlhe40 + siZbp1 group was significantly lower than in the Bhlhe40 + siNC group (Figure 7J,K). These results demonstrated that BHLHE40 maintains the stemness of PαS cells by activating *Zbp1*.

### 3.7. BHLHE40 Regulates the Stemness of PαS Cells through the Wnt/β-Catenin Signaling Pathway

**Figure 8:**
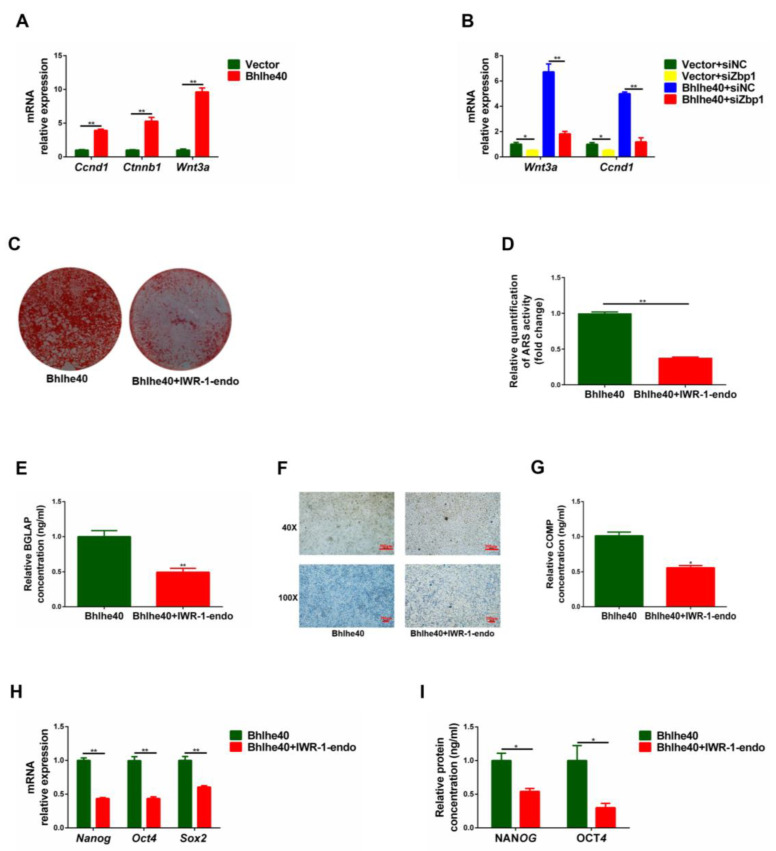
BHLHE40 regulates the stemness of PαS cells through Wnt/β-catenin signaling pathway. (**A**) Relative expression of *Ccnd1*, *Ctnnb1*, and *Wnt3a* mRNA was evaluated by qRT-PCR in overexpressed *Bhlhe40* (Bhlhe40) group and negative control (Vector) group in vitro. *Gapdh* was used for normalization. (**B**) Relative expression of *Wnt3a*, and *Ccnd1* mRNA was evaluated by qRT-PCR in Bhlhe40 group and Vector group transfected with si*Zbp1* and siNC, respectively. *Gapdh* was used for normalization. (**C**,**D**) ARS staining and quantification of Bhlhe40 group and Bhlhe40 + IWR-1-endo group in vitro after 14 days of osteogenic differentiation. (**E**) Relative expression of BGLAP protein was evaluated by ELISA in Bhlhe40 group and Bhlhe40 + IWR-1-endo group in vitro after 14 days of osteogenic differentiation. (**F**) Alcian blue staining in well plates of Bhlhe40 group and Bhlhe40 + IWR-1-endo group after 21 days of chondrogenic differentiation. Scale bar = 100 μm and 500 μm, *n* = 3. (**G**) Relative expression of COMP protein was evaluated by ELISA in Bhlhe40 group and Bhlhe40 + IWR-1-endo in vitro after 21 days of chondrogenic differentiation. (**H**) Relative expression of *Nanog*, *Oct4*, and *Sox2* mRNA was evaluated by qRT-PCR in Bhlhe40 group and Bhlhe40+IWR-1-endo group in vitro. *Gapdh* was used for normalization. (**I**) Relative expression of NANOG and OCT4 protein was evaluated by ELISA in Bhlhe40 + IWR-1-endo group and Vector group in vitro. Data are represented as mean ± SD of three independent experiments. * *p* < 0.05; ** *p* < 0.01, Student’s *t*-test and one-way ANOVA. BHLHE40, basic helix–loop–helix family member E40; *Wnt3a*, wingless-type MMTV integration site family, member 3A; *Ctnnb1*, cadherin associated protein, beta 1; *Ccnd1*, cyclin D1; ARS, alizarin red S; *Gapdh*, glyceraldehyde 3-phosphate dehydrogenase; si*Zbp1*, small interfering Z-DNA binding protein 1; siNC, short interfering negative control; qRT-PCR, quantitative reverse transcription polymerase chain reaction; ELISA, enzyme linked immunosorbent assay; BGLAP, bone gamma carboxyglutamate protein; COMP, cartilage oligomeric matrix protein; *Nanog*, nanog homeobox; *Sox2*, sex determining region Y-box 2; OCT4, organic cation/carnitine transporter4; ANOVA, analysis of variance.

Studies have reported that *Zbp1* regulates the Wnt/β-catenin signaling pathway to promote osteogenesis, so we subsequently explored the function of the Wnt/β-catenin signaling pathway in this study [43,44]. The mRNA expression of key genes (*Wnt3a*, *Ctnnb1*, and *Ccnd1*) in the Wnt/β-catenin signaling pathway displayed that the Bhlhe40 group was significantly higher than the Vector group (Figure 8A). Moreover, *Wnt3a* and *Ccnd1* were significantly higher in the Bhlhe40 + siZbp1 group than in the Bhlhe40 + siNC group (Figure 8B). These results indicated that BHLHE40 may activate Wnt/β-catenin signaling in PαS cells.

After 14 days of osteogenic induction, the ARS staining and quantitative results showed that the Bhlhe40 + IWR-1-endo group was significantly less than the Bhlhe40 group (Figure 8C,D). In addition, the ELISA results of BGLAP showed a similar trend (Figure 8E). After 21 days of chondrogenic induction, the Alcian blue staining in well plate results and the ELISA results of COMP both displayed that the Bhlhe40 + IWR-1-endo group was significantly less than the Bhlhe40 group (Figure 8F,G). Furthermore, qRT-PCR and ELISA results displayed that the expression of stemness genes in the Bhlhe40 + IWR-1-endo group was significantly lower than in the Bhlhe40 group (Figure 8H,I). These results demonstrated that BHLHE40 regulates the stemness of PαS cells through the Wnt/β-catenin signaling pathway.

## 4. Discussion

The age of a stem cell donor has a significant effect on the number and stemness of the cells obtained [45,46,47]. A previous study compared the proportions of PαS cells found in the long bones of C57/6N mice at four different stages of development and found that the highest proportions of PαS cells were obtained from the limbs of C57/6N mice at embryonic day 18.5 and postnatal days 1 to 3 [30]. Therefore, considering the difficulty of the experimental procedure, the experimental cost, and the number of cells extracted, C57/6N mice at postnatal day 1 to 3 were selected for the extraction of fresh PαS cells in this study.

Previous studies have investigated stemness changes during in vitro culture, whereas few studies have focused on differences in stemness between stem cells that have been freshly collected from the body and those that have been cultured in vitro [48,49]. In this study, fresh PαS cells were isolated prospectively by FACS, and we compared the stemness of fresh and P0 PαS cells. We found that fresh PαS cells once adherent cultured in vitro, show some irregular differentiation tendency. This further illustrates the importance of establishing safe and effective amplification methods in vitro. However, the fresh PαS cells obtained by FACS in our current work were not identical to real PαS cells in vivo. Implementing a lineage tracing experiment for PαS cells in mice may help to address this problem [50].

In this study, flow cytometry displayed that the expression of surface markers was significantly higher in fresh PαS cells than in P0 PαS cells. These results also suggest that the characteristics of fresh PαS cells change considerably when the cells are cultured in vitro. Few previous studies have noted that markers are lost from the surface of PαS cells cultured in vitro. H&E staining of ectopic osteogenesis in vivo showed more lamellar bone tissue in fresh PαS cells than in P0 PαS cells. Our results also confirmed that there are many differences in the biological characteristics of BMSCs in vivo, compared to BMSCs that have been cultured in vitro [7,8]. As far as we know, no research has reported on differences in stemness between PαS cells freshly sorted representative of in vivo and PαS cells cultured in vitro. In addition, we found that the stemness of fresh PαS cells sorted representative of in vivo was superior to that of P0 PαS cells cultured in vitro. The results of this part also suggest that in order to maintain the characteristics of stem cells, more attention needs to be paid to the changes of stem cells from in vivo to in vitro nodes to obtain a better effect of stem cell therapy.

The PαS cells used in this study were only cultured in vitro to P4, because the growth of these PαS cells was very slow, and the differentiation potential of these cells was quite weak. In addition, the cell proliferation and differentiation capacities of PαS cells changed significantly from P0 to P4. Therefore, we only investigated changes in the proliferation and differentiation capacities of PαS cells from P0 to P4. Importantly, we noted that the proliferation and differentiation capacities of PαS cells decreased considerably from P0 to P1 in vitro, and this was confirmed by in vivo ectopic osteogenesis experiments (Figure 3A). H&E staining of P0 PαS cells clearly identified lamellar bone tissue, whereas P1 PαS cells did not. These results suggest that we should pay more attention to the biological changes of PαS cells from P0 to P1 in vitro.

Our flow cytometry and mRNA expression results displayed that the expression of surface markers in PαS cells in vitro decreased gradually from P0 to P4. These results demonstrated that the stemness of PαS cells decreased gradually due to the loss of some of their original characteristics during in vitro culture. In addition, these results were also consistent with the above trend of reduced proliferation and differentiation ability of PαS cells from P0 to P4 in vitro. Previous studies reported that SCA-1^+^ stem cells have a higher proliferative capacity and greater osteogenic differentiation capacities than SCA-1^−^ stem cells [51]. Therefore, the decreased stemness of PαS cells may be linked to decreased expression of the SCA-1 surface marker. Even if the stemness of PαS cells in vitro is linked to the expression of markers on the cell surface, further studies will be needed to elucidate the relevant mechanisms.

Of course, there are still some limitations of this study. First of all, overexpression of *Bhlhe40* may cause a ripple reaction, which can lead to some unexpected effects on the PαS cells. In addition, the animal model of stemness research in vivo is relatively simple, and this animal research model can be enriched in the future when conditions are available. Furthermore, this study did not explore what regulates the transcription factor BHLHE40, which will be our next step. Finally, due to time and money constraints, this study did not use a transgenic mouse to further confirm the role of BHLHE40 in vivo, which is also the focus of our future research work.

This present work first investigated the pivotal role of BHLHE40 in maintaining the stemness of PαS cells in vitro, and demonstrated that BHLHE40 activated the Wnt/β-catenin signaling pathway in PαS cells by targeting *Zbp1*. These data provide a basis for the development of small molecule drugs that target BHLHE40 or stimulate BHLHE40 expression. A better understanding of the properties of freshly isolated stem cells and how to optimize the regulation of stem cell differentiation during in vitro expansion will inform practitioners and help to alleviate the bottleneck of clinical application.

## 5. Conclusions

Our study indicated that the stemness of fresh PαS cells representative in vivo was superior to that of P0 PαS cells in vitro. In addition, the stemness of PαS cells decreased gradually from P0 to P4 in vivo and in vitro. Moreover, BHLHE40 maintained the stemness of PαS cells by targeting *Zbp1* through the Wnt/β-catenin signaling pathway in vitro. This work may provide significant indications to improve the clinical applications of PαS cells in the future.

## Figures and Tables

**Table 2:** BHLHE40 may bind to candidate target genes related to stemness.

Gene	Chr	Score of Significance	Region
*Chd7*	chr4	406.97581	genebody
*Mettl3*	chr14	26.26893	promoter
*Foxo3*	chr10	352.03472	promoter
*Foxp1*	chr6	84.28053	promoter
*Kdm4c*	chr4	28.58553	genebody
*Kdm3a*	chr6	23.25266	promoter
*Kdm2b*	chr5	27.44679	promoter
*Zbp1*	chr2	76.28664	promoter
*Kdm6a*	chrX	83.51532	promoter
*Kdm1a*	chr4	2.56084	promoter
*Cdk6*	chr5	3.24094	promoter
*P2rx7*	chr5	26.09183	promoter
*Hoxc10*	chr15	29.97397	promoter
*Smad3*	chr9	105.85294	promoter
*Hdac4*	chr1	106.92581	promoter
*Tet2*	chr3	210.94673	promoter
*Sirt1*	chr10	164.0141	promoter

## Data Availability

All data are included in the manuscript and supporting information. The datasets generated during and/or analyzed during the current study is available from the corresponding author on reasonable request.

## Supplementary Materials

The following supporting information can be downloaded at: https://www.mdpi.com/article/10.3390/biomedicines11082190/s1, Table S1: The specific information of animals, materials, and reagents used in this study.
