# Supplementary material for: BHLHE40 Maintains the Stemness of PαS Cells In Vitro by Targeting Zbp1 through the Wnt/β-Catenin Signaling Pathway
Source: Biomedicines. 2023 Aug 3;11(8):2190. doi: 10.3390/biomedicines11082190 (PMC10452820; doi:10.3390/biomedicines11082190)
Supplement: Supplementary file 1 [file biomedicines-11-02190-s001.zip › biomedicines-2493407-supplementary.pdf]

Supplementary Materials

Table S1. The specific information of animals, materials, and reagents used in this study.

Animals/Reagents/Materials	Company	City	Country
C57BL/6N	Charles River	Beijing	China
BALB/c nude mice	Charles River	Beijing	China
Cell culture materiasl	Corning	Corning	USA
β-TCP	Bicon	Boston	USA
collagen membrane	Bonag	Beijing	China
α-MEM	Gibco	Grand island	USA
FBS	Gibco	Grand island	USA
penicillin G	Gibco	Grand island	USA
streptomycin	Gibco	Grand island	USA
EDTA	Gibco	Grand island	USA
dexamethasone	Sigma	St. Louis	USA
L-ascorbic acid	Sigma	St. Louis	USA
β-glycerophosphoric acid	Sigma	St. Louis	USA
L-lactic acid diphosphate	Sigma	St. Louis	USA
sodium pyruvate	Boaotoda Technology	Beijing	China
L-proline	Boaotoda Technology	Beijing	China
TGF-β1	Nearshore Protein Technology	Suzhou	China
ITS	Biyuntian	Shanghai	China
Bhlhe40-overexpressing lentivirus (Bhlhe40)	GenePharma	Suzhou	China
negative control lentivirus (Vector)	GenePharma	Suzhou	China
polystyrene	Sigma	St. Louis	USA
puromycin	Sigma	St. Louis	USA
si*Zbp1*	GenePharma	Suzhou	China
siNC	GenePharma	Suzhou	China
Lipofectamine 3000	Invitrogen	Carlsbad	USA
IWR-1 endo	Maclean Biochemical Technology	Shanghai	China
CCK8	Dojindo	Kumamoto	Japan
PBS	Sigma	St. Louis	USA
paraformaldehyde	Sigma	St. Louis	USA
crystal violet	Puli Gene Technology	Beijing	China
ALP Staining Kit	Biyuntian	Shanghai	China
BCA protein detection kit	Thermo Scientific	Waltham	USA
ALP detection kit	Jiancheng Bioengineering Institute	Nanjing	China
ARS solution	Sigma	St. Louis	USA
cetylpyridine chlorination	Sigma	St. Louis	USA
Alcian blue solution	Solarbio	Beijing	China
TRIzol	Invitrogen	Carlsbad	USA
Prime Script RT Reagent Kit	Takara	Tokyo	Japan
Power SYBR Green PCR Master Mix	Roche Applied Science	Mannheim	Germany
CD45 antibody	BioLegend	San Diego	USA
PDGFRα antibody	BioLegend	San Diego	USA
TER119 antibody	BioLegend	San Diego	USA

SCA-1 antibody	BioLegend	San Diego	USA
BGP ELISA kit	Illarite Biotechnology	Wuhan	China
PPARγ ELISA kit	Illarite Biotechnology	Wuhan	China
COMP ELISA kit	Cloud Clone Technology	Wuhan	China
NANOG ELISA kit	Cloud Clone Technology	Wuhan	China
OCT4 ELISA kit	Cloud Clone Technology	Wuhan	China
BHLHE40 IP antibody	Proteintech Group	San Diego	USA
ChIP kit	Cell Signaling Technology	Boston	USA
